# Cognitive chicken or the emotional egg? How reconceptualizing decision-making by integrating cognition and emotion can improve task psychometrics and clinical utility

**DOI:** 10.3389/fpsyg.2023.1254179

**Published:** 2023-11-16

**Authors:** Melissa T. Buelow, Sammy Moore, Jennifer M. Kowalsky, Bradley M. Okdie

**Affiliations:** ^1^Department of Psychology, The Ohio State University, Newark, OH, United States; ^2^School of Psychological Sciences, The University of Western Australia, Perth, WA, Australia

**Keywords:** decision-making, emotion, cognition, executive function, validity

## Abstract

Decision-making is an executive function, tapping into cognitive, emotional, and personality-based components. This complexity, and the varying operational definitions of the construct, is reflected in the rich array of behavioral decision-making tasks available for use in research and clinical settings. In many cases, these tasks are “subfield-specific,” with tasks developed by cognitive psychologists focusing on cognitive aspects of decision-making and tasks developed by clinical psychologists focusing on interactions between emotional and cognitive aspects. Critically, performance across different tasks does not consistently correlate, obfuscating the ability to compare scores between measures and detect changes over time. Differing theories as to what cognitive and/or emotional aspects affect decision-making likely contribute to this lack of consistency across measures. The low criterion-related validity among decision-making tasks and lack of consistent measurement of the construct presents challenges for emotion and decision-making scholars. In this perspective, we provide several recommendations for the field: (a) assess decision-making as a specific cognitive ability versus a taxonomy of cognitive abilities; (b) a renewed focus on convergent validity across tasks; (c) further assessment of test–retest reliability versus practice effects on tasks; and (d) reimagine future decision-making research to consider the research versus clinical implications. We discuss one example of decision-making research applied to clinical settings, acquired brain injury recovery, to demonstrate how some of these concerns and recommendations can affect the ability to track changes in decision-making across time.

## 1. Introduction

Decision-making belongs to a group of higher-order, complex cognitive abilities linked to the prefrontal cortex (PFC) (Lezak et al., 2004) known as executive functions (EF). Decisions that are more emotion-based often activate ventromedial prefrontal cortex (VMPFC) (Ernst et al., 2002; Cho et al., 2012; Paulsen et al., 2012), whereas decisions that are more cognition-based activate dorsolateral prefrontal cortex (DLPFC) (Krain et al., 2006; Rolls and Grabenhorst, 2008). Yet both VMPFC and DLPFC can contribute to either type of decision-making. The brain’s reward pathway links the PFC with the limbic system and midbrain (Glimcher et al., 2009; Galvan, 2012), and decision-making activates this pathway (Jessor, 1991; Elliott et al., 2008; Curtis and Lee, 2010; Reyna, 2012). But aspects of the limbic system (Dalgleish, 2004; Fossati, 2012) and PFC (Baker et al., 1997) are linked to the processing of emotions, demonstrating that cognition and emotion utilize similar circuitry. Should we disentangle cognition and emotion in decision-making, or do we need to consider these two components together when defining the construct? It is possible that keeping emotion out of cognition (and vice versa) when defining decision-making has led to some of the current issues affecting the field. In the following sections, we provide a review of the current status of several important issues for decision-making as an individual differences variable and provide recommendations for the field moving forward. As applicable, we discuss where there may be different needs for those in the research versus clinical realms.

## 2. Specificity versus taxonomy of decision-making

Although a full review of the history of decision-making, and all the relevant theories of it, is outside the scope of this perspective, several reviews and key pieces are available regarding signal detection theory (Stanislaw and Todorov, 1999), expected value- and utility-based theories (Von Neumann and Morgenstern, 1944; Machina, 1987), prospect theory (Kahneman and Tversky, 1979), and dual (Tversky and Kahneman, 1983; Metcalfe and Mischel, 1999; Reyna, 2004; Evans, 2008) and triple (Wood and Bechara, 2014) process system theories of decision-making. A key component of several of these theories is the extent to which utilizing cognitive factors (e.g., working memory, knowledge of probabilities, abstract reasoning; Reyna et al., 2009; Weber and Johnson, 2009; Curtis and Lee, 2010; Reyna and Brainerd, 2011; Brand et al., 2014) and emotional factors (Suhr and Tsanadis, 2007; Roiser et al., 2009; Buelow et al., 2013) can affect decisions. A differing emphasis on emotion versus cognition can lead to differences in the overall conceptualization of decision-making and, subsequently, in how it is assessed.

Is decision-making a specific cognitive ability, or does it instead represent a taxonomy of related cognitive abilities that are tied to the PFC, limbic system, and their subcortical connections? Is decision-making similar to the other executive functions, in that it is made up of different aspects of the construct rather than one overarching ability that is applied in every situation? To the extent that a decision is made in a consistent manner, across situations and across time, then decision-making may be more of a specific cognitive ability. Yet as many show (e.g., Schoemaker, 1993; Ert and Yechiam, 2010; Figner and Weber, 2011; Yechiam and Ert, 2011), individuals do not consistently weigh gains and losses (or risks and benefits) across situations. How questions are worded/framed (e.g., gain-framing versus loss-framing; Tversky and Kahneman, 1981; Yechiam and Ert, 2011) can lead to different decisions. These inconsistencies may also occur based on the specific type of risk, the extent to which cognitive and emotional factors come into play, and one’s own risk-taking propensity (e.g., Figner and Weber, 2011). These inconsistencies lead us to wonder if decision-making mimics executive functions, in that it serves as a taxonomy for a series of ‘subcomponents’ that make up the overarching construct. Several of these subcomponents may be activated in one situation but not in another, which could account for the inconsistencies when performance is assessed across tasks. Moving forward, the field should consider the overarching construct of decision making and whether it is a specific ability or a higher-order classification for a set of cognitive abilities.

## 3. Convergent validity across tasks

If decision-making is a specific cognitive ability, and did not represent a taxonomy like the executive functions, then tasks assessing decision-making should show strong convergent validity. A task such as the Iowa Gambling Task (IGT; Bechara et al., 1994) should show strong correlations with the Balloon Analog Risk Task (BART; Lejuez et al., 2002), Game of Dice Task (GDT; Brand et al., 2007), and others. Some previous research finds such correlations (Brand et al., 2007; Henninger et al., 2010; Koritzky and Yechiam, 2010; Upton et al., 2011; MacKillop et al., 2014; Brown et al., 2015), but most instead show weak/small (Skeel et al., 2007; Mäntylä et al., 2012; Brunell and Buelow, 2017) or no (Mäntylä et al., 2012; Pletzer and Ortner, 2016) correlations between decision-making tasks designed to assess individual differences in the construct. If decision-making instead represents a taxonomy of cognitive abilities, then these inconsistencies make sense. Tasks that pull for more “cold” executive functions may correlate more with each other than with tasks that pull more for “hot” executive functions. We see this relationship when assessing correlations between the Wisconsin Card Sort Task, a measure of cold EF linked to the DLPFC (Lezak et al., 2004), as it correlates with decision-making tasks assessing more cold cognitions (e.g., Columbia Card Task, GDT; Brand et al., 2007, 2014; Buelow, 2015) but not with more hot/emotion-based tasks (e.g., IGT; Bechara et al., 2001; Reynolds et al., 2019). Factor analyses also demonstrate this lack of convergent validity (Buelow and Blaine, 2015).

Research investigating cognitive models of decisions on behavioral tasks point toward a better understanding of decision-making and convergent validity across tasks. Previous research points to factors such as sensitivity to gains/rewards (Yechiam and Busemeyer, 2008; Ert and Yechiam, 2010; Brevers et al., 2014), sensitivity to losses/risks (Bishara et al., 2009; Ert and Yechiam, 2010), frequency of gains and losses (Lin et al., 2009), choice consistency (Stout et al., 2004; Yechiam et al., 2005; Lin et al., 2016), discounting of versus learning from feedback (Yechiam et al., 2005; Prause and Lawyer, 2014; Byrne and Worthy, 2016), and individual differences in risk perception and acceptance (Wallsten et al., 2005; Ert and Yechiam, 2010; Figner and Weber, 2011; Yechiam and Ert, 2011) can affect decision-making across multiple tasks. It is possible that the relative lack of convergent validity across tasks to date is partly due to the nature of decision-making itself (e.g., specificity versus taxonomy) and partly due to the use of total/net scores to assess decision-making. Future research should continue to investigate these cognitive modeling-based commonalities across tasks, as there may be more convergence than it appears.

## 4. Test–retest reliability

How does performance on decision-making tasks change across time? Is decision-making a relatively stable construct, or does it change across time or based on other factors? To assess the stability of decision-making, we can assess the test–retest reliability of various behavioral tasks. Estimates of test–retest reliability vary across specific tasks, samples, and time periods, but overall there are often moderate to strong correlations between tasks administered days (Johnson and Bruner, 2013; Weafer et al., 2013), weeks (Buelow and Barnhart, 2018), months (Forster et al., 2016; Peng et al., 2018), and years (Kirby, 2009; Yechiam and Ert, 2011) apart. Although these correlations are relatively high, interpretation of them as evidence in favor of strong test–retest reliability depends on the context. Portney and Watkins (2015)‘s guidelines for clinical measures are that correlations across time of 0.50–0.75 are poor/moderate while those over 0.75 are acceptable. Few—if any—of these correlations meet that criterion. In addition, there are some tasks that are single-use, as there is some element (e.g., learning the risks/benefits of the decks on the IGT) that cannot be ‘unlearned’ to allow for a second, future administration. On the IGT in particular, evidence of these learned practice effects is evident even years after the initial administration (e.g., Waters-Wood et al., 2012; see Buelow, 2020, for review). Although lab-based studies of healthy control or clinical participants may require a one-time assessment of current decision-making skills, real-world clinical evaluations can require multiple assessments over time. Tracking decision-making over time is difficult when (a) tasks lack test–retest reliability and convergent validity and (b) there are no clinically-available tasks (i.e., those that have been validated in clinical samples and for which normative data is available) that can be used in a longitudinal assessment. As we will discuss in the next section, concerns about reliability and validity may differ based on the implications of a research study.

## 5. Reliability, validity, and experimental designs

As the field of decision science moves forward, the noted concerns about reliability and validity can point in different directions for new experimental designs, in part due to the basis for or reasoning behind a new research study. Is the intent to predict behavior? Document impaired decision-making based on a situational factor, individual differences factor, or psychological or medical/neurological diagnosis? Assess potential for change in the future? Understand decision-making or other cognitive/emotional process? Although in some cases it may be sufficient to document a one-time only assessment of decision-making impairment due to a diagnosis or other factor, in other cases the findings may have implications for treatments to improve decision-making skills among, for example, those with an acquired brain injury.

Acquired brain injury (ABI) is one example where more research is needed into both test–retest reliability and convergent validity, as tracking changes in cognitive difficulties across time is important for the patient with an ABI. ABI refers to any injury to the brain, such as what occurs in the course of traumatic (TBI; e.g., from accidents or falls) or nontraumatic (e.g., stroke) injury (Cullen et al., 2008). Although cognitive and emotional consequences of ABI vary due to the specific regions affected, the PFC is often negatively impacted (McAllister, 2011). Following ABI, patients may experience difficulties with attention, memory encoding and retrieval, processing speed, and EF (Allanson et al., 2017; Watson et al., 2020); however, these cited meta-analyses do not assess how hot EFs, such as decision-making, change post-ABI. Many clinically-available EF tasks focus on cold cognitive processes, even though OFC, VMPFC, and medial PFC may also be affected. Results of EF assessments predict long-term outcome for patients post-ABI (Allanson et al., 2017), yet this is often predicated on results of cold (non-emotion based) EF assessment. Individuals with ABI can also experience changes in emotional experience and expression (Gouick and Gentleman, 2004; de Sousa et al., 2012; with Phineas Gage as an early example).

In their Jose et al. (2020) review, Jose and colleagues examined the current understanding of EF in ABI. They divided decision-making into a set of skills that include value coding, social context, and emotional dysregulation, with moral reasoning and working memory as related cognitive functions. Research suggests that VMPFC, DLPFC, and OFC are involved in each of these decision-making components, despite the belief that each structure is specific to one cognitive (or emotional) function. They point out that focusing on a specific/small area of cognition can result in a loss of the bigger picture in ABI, such as by focusing on value coding one loses sight of the working memory and emotion regulation difficulties that could also affect decision-making.

Following ABI, individuals are typically assessed in the acute recovery phase to (a) predict pre-morbid functioning; (b) determine their strengths and weaknesses post-injury; and (c) develop a treatment plan. This gives a single snapshot of that individual’s cognitive history, yet it is often used for future comparison. Although important to identify cognitive deficits, it is also important to document if those deficits resolve. The IGT is currently the only behavioral task with available normative data to guide interpretation of task performance in clinical populations (Bechara, 2007), with a recent meta-analysis supporting its utility to assess decision-making impairments post-ABI (regardless of lesion location; Moore et al., 2023, under review)^1^. However, even 1+ years after initial assessment, practice effects remain (not specific to ABI samples; Tuvblad et al., 2013; Xiao et al., 2013). If there were additional tasks that demonstrated strong convergent validity with the IGT, they could instead be used to assess decision-making change over time. But there is a lack of convergent validity across decision-making tasks, creating difficulty showing improved decision-making as individuals recover from ABI. And while there is a typical recovery period post-ABI, especially from traumatic etiologies (Cullen et al., 2008), our inability to track decision-making changes across time limits what we can learn about EF recovery and how decision-making strategies may change post-ABI.

Outside of ABI and other clinical diagnoses where repeatable decision-making tasks can be of benefit, within-subjects study designs can also benefit from repeatable tasks. As just one example, multiple studies have shown that participants’ current mood or emotional state can affect decision-making (e.g., Mitchell and Phillips, 2007; Tamir and Robinson, 2007; Buelow et al., 2013; Forgas, 2013). In most of the lab-based manipulations of mood, decision-making was assessed post-manipulation only. If there was a repeatable decision-making task, it could be utilized in a pre-post manipulation, within-participant study design. This would additionally allow researchers to assess the extent to which the mood manipulation interacted with prior characteristics of the individual and their decision-making ability to affect subsequent performance.

## 6. Conclusion

The cognitive and emotional conceptualizations of decision-making need to be more precise: when should cognition and emotion be integrated, and when should they be assessed separately? Applications of dual process theory suggest cognition and emotion inform each other, but many current tasks rely on operational definitions of decision-making focused on only cognition or only emotion. Emotion can inform our understanding of cognition, and cognition can inform our understanding of emotion. VMPFC and DLPFC pathways are functionally connected, forming a prefrontal-cingulate cortex network that combines elements of hot and cold (“hot-cold”) decision-making (Jose et al., 2020; Salehinejad et al., 2021). Even tasks designed to examine emotion-based decision-making are correlated with measures of EFs and other cognitive abilities, indicating you cannot take cognition out of emotion either. Continuing to ‘silo’ cognitive and emotional theories of decision-making limits our ability to understand why individuals take risks and make suboptimal decisions. Utilizing an integrated conceptualization of decision-making and better centering it within other EFs may help researchers develop new tasks with adequate psychometrics that can be used to track recovery from an acute TBI, for example.

We believe the field of decision science still has much to offer our understanding of decision-making processes. However, the field’s impact is constrained by the lack of good psychometrics for many tasks and inconsistencies in conceptualizations of decision-making leading to differences in explaining task performance. The incorporation of cognitive and affective components of decision-making into a single taxonomy will methodologically increase reliability and validity of decision-making measures, as tasks born out of this conceptualization should, at their core, be assessing an overarching conceptualization that includes the assessment of both cognitive and affective components of decision-making.

## Author contributions

MB: Conceptualization, Writing – original draft, Writing – review & editing. SM: Conceptualization, Writing – review & editing. JK: Conceptualization, Writing – review & editing. BO: Conceptualization, Writing – review & editing.

## Footnotes

^1^Moore, S., Naragon-Gainey, K., Pestell, C.F., Becerra, R., Buelow, M.T., and Fynn, D.M. (2023). The level and nature of impairment on the Iowa gambling task following acquired brain injury: a meta-analysis. (under review).
